# Hetero Nucleus Growth Stabilizing Zinc Anode for High-Biosecurity Zinc-Ion Batteries

**DOI:** 10.1007/s40820-023-01206-2

**Published:** 2023-10-26

**Authors:** Jingjing Li, Zhexuan Liu, Shaohua Han, Peng Zhou, Bingan Lu, Jianda Zhou, Zhiyuan Zeng, Zhizhao Chen, Jiang Zhou

**Affiliations:** 1 Department of Plastic Surgery and National Clinical Research Center for Geriatric Disorders, Xiangya Hospital, Central South University, Changsha, 410008 People’s Republic of China; 2 School of Materials Science and Engineering, Hunan Provincial Key Laboratory of Electronic Packaging and Advanced Functional Materials, Central South University, Changsha, 410083 People’s Republic of China; 3 Hunan Provincial Key Defense Laboratory of High Temperature Wear-Resisting Materials and Preparation Technology, Hunan University of Science and Technology, Xiangtan, 411201 People’s Republic of China; 4 School of Physics and Electronics, Hunan University, Changsha, 410082 People’s Republic of China; 5 Department of Plastic Surgery, The Third Xiangya Hospital, Central South University, Changsha, 410013 People’s Republic of China; 6 Department of Materials Science and Engineering, City University of Hong Kong, 83 Tat Chee Avenue, Kowloon, 999077 Hong Kong People’s Republic of China

**Keywords:** Aqueous zinc-ion batteries, Biocompatible devices, Operating stability, Zinc anode, Zinc salts electrolyte

## Abstract

Biocompatible devices are widely employed in modernized lives and medical fields in the forms of wearable and implantable devices, raising higher requirements on the battery biocompatibility, high safety, low cost, and excellent electrochemical performance, which become the evaluation criteria toward developing feasible biocompatible batteries. Herein, through conducting the battery implantation tests and leakage scene simulations on New Zealand rabbits, zinc sulfate electrolyte is proved to exhibit higher biosecurity and turns out to be one of the ideal zinc salts for biocompatible zinc-ion batteries (ZIBs). Furthermore, in order to mitigate the notorious dendrite growth and hydrogen evolution in mildly acidic electrolyte as well as improve their operating stability, Sn hetero nucleus is introduced to stabilize the zinc anode, which not only facilitates the planar zinc deposition, but also contributes to higher hydrogen evolution overpotential. Finally, a long lifetime of 1500 h for the symmetrical cell, the specific capacity of 150 mAh g^−1^ under 0.5 A g^−1^ for the Zn–MnO_2_ battery and 212 mAh g^−1^ under 5 A g^−1^ for the Zn—NH_4_V_4_O_10_ battery are obtained. This work may provide unique perspectives on biocompatible ZIBs toward the biosecurity of their cell components.

**Supplementary Information:**

The online version contains supplementary material available at 10.1007/s40820-023-01206-2.

## Highlights

- Animal models are applied to evaluate the biosecurity and biocompatibility of the zinc-ion batteries with the electrolytes of different zinc salts.
- Leakage scene simulations and histological analysis are employed in investigating the tissue response after battery implantations, in which ZnSO_4_ exhibits higher biosecurity.
- Sn hetero nucleus is introduced to stabilize the zinc anode, which not only facilitates the planar zinc deposition, but also contributes to higher hydrogen evolution overpotential.

**Supplementary Information:**

The online version contains supplementary material available at 10.1007/s40820-023-01206-2.

## Graphical abstract

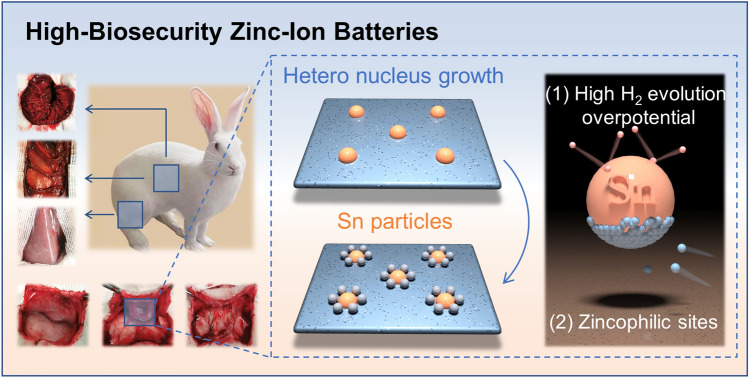

## Introduction

Thanks to the rapid development of advanced techniques and theories, biocompatible devices are applied in various aspects of human lives in these years, of which the biocompatibility is significant when evaluating their feasibility [1, 2]. Biocompatible devices generally include wearable and implantable electronics, depending on their operating environments [3]. Wearable electronics refer to the electronic devices that can be worn or pasted on the body, which are involved in many aspects of cutting‐edge research in the fields of smartwatches, fitness trackers, smart clothing sensors, and Internet of Things [4–7]. With the increasing demand for wearable electronics, biocompatible and reliable power source with eco-friendly, low-cost, and multifunctional characteristics are imperative to be constructed [8, 9]. Implantable devices are of great potential in medical fields, including cardiac pacemakers, cardioverter-defibrillator, total artificial hearts, implantable nerve stimulators, cochlear implants, implantable bone growth stimulators, and implantable drug pumps [10, 11]. Limited by the incommodity of surgical removal, the long-term availability is vital for most biocompatible devices, thus delivering higher demands on the biocompatible batteries, which provide the required energy for the whole device [12, 13]. Among the required properties of wearable and implantable batteries, biocompatibility should be preferentially considered, including operating stability and biosecurity.

Basically, aqueous energy storage techniques exhibit tremendous advantages for powering these electronics [14–16]. Among them, zinc-ion batteries (ZIBs) have attracted much attentions in relevant energy storage field due to their excellent stability and low cost [17–20]. Based on the aqueous electrolyte, ZIBs have been selected as the candidates for powering biocompatible electronics [3, 21, 22]. On the one hand, substantial efforts have been made on biocompatible ZIBs and their operating stability because of their high safety, intrinsic inertness, and compatibility with hydrogel electrolytes [23–26]. Relevant research leads to gratifying results, as various battery configurations have been implemented, including cable type [27], planar type [28], stack type [29], etc. On the other hand, high biosecurity ZIBs are especially potential prospect, as zinc is a naturally occurring element in the body and is essential for proper functioning [30]. Unfortunately, there are still few reports investigating the biosecurity of ZIBs, not to mention the corresponding experimental validation [31, 32]. Therefore, more efforts should be focused on the biosecurity of biocompatible ZIBs, while their long-term operating stability should be promised as well [33].

**Fig. 1:**
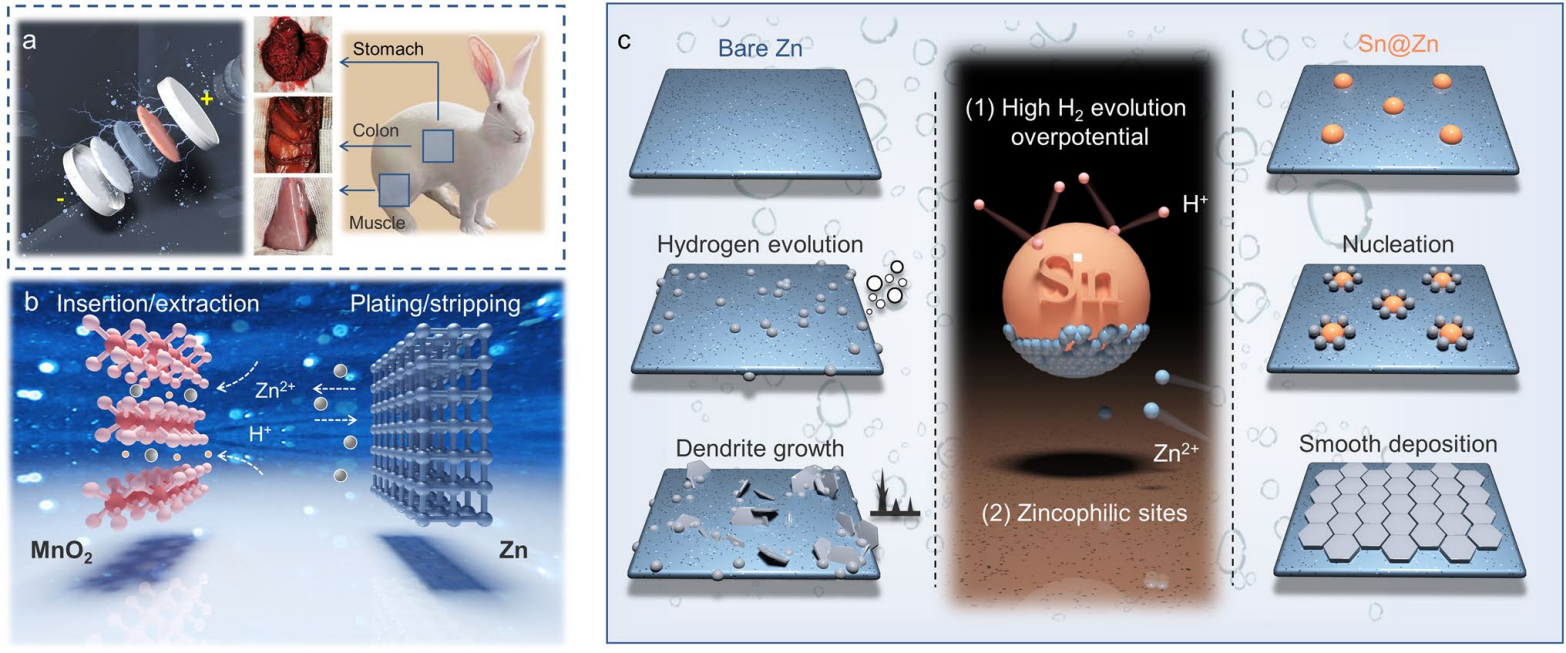
An overview of the relevant related works. **a** Implantable batteries and the experimental animal models. **b** Working mechanism of the Zn–MnO_2_ batteries. **c** Schematics of introducing the Sn hetero nucleus and its effects on mitigating dendrite growth and hydrogen evolution reaction

Herein, through conducting the battery implantation tests and leakage scene simulations on New Zealand rabbits (Fig. 1a), aqueous zinc salt electrolytes are proved to exhibit higher biosecurity than organic lithium salt electrolyte. Importantly, ZnSO_4_ turns to be one of the ideal zinc salts for biocompatible ZIBs, which are based on the cathodic insertion/extraction and anodic plating/stripping (Fig. 1b). Except for high biosecurity, operating stability is also significant for biocompatible ZIBs. Thus, in order to mitigate the notorious dendrite growth and hydrogen evolution in mildly acidic electrolyte [34, 35], which may induce battery inflation and short circuit [36], Sn hetero nucleus is introduced to modify the zinc foil surface. This Sn@Zn anode not only facilitates the planar zinc deposition, but also be endowed with higher hydrogen evolution overpotential (Fig. 1c), leading to much lower polarization voltage gap and longer lifetime in symmetrical cells. Coupled with MnO_2_ and NH_4_V_4_O_10_ cathode materials, the batteries exhibit high specific capacities of 150 mAh g^−1^ under 0.5 A g^−1^ (300 cycles) and 212 mAh g^−1^ under 5 A g^−1^ (1000 cycles), respectively, presenting desirable electrochemical performance.

## Experimental Section

### Materials

All the reagents are of analytical purity and used as received without further purification. Zinc sulfate heptahydrate (ZnSO_4_·7H_2_O, ≧ 99%), stannous sulfate (SnSO_4_, ≧ 98%), manganese sulfate (MnSO_4_, ≧ 99%), zinc acetate tetrahydrate (Zn(CH_3_COO)_2_·4H_2_O, ≧ 99%), zinc trifluoromethanesulfonate (Zn(CF_3_SO_3_)_2_, ≧ 99%), and lithium hexafluorophosphate (LiPF_6_/EC, 5%) are used.

### Synthesis of the Materials

#### Construction of Sn@Zn Foil

About 0.01 M SnSO_4_ solution is obtained by dissolving SnSO_4_ into deionized water and string for 30 min. Bare Zn foil is cut into needed circular sheets (15-mm diameter) and immersed in 0.01 M SnSO_4_ solution for 1 min. The obtained Sn@Zn foil is washed with deionized water for several times and dried at 80 °C in air for 12 h.

#### ***Synthesis of CNT@MnO***_***2***_

About 1.5-g multiwalled carbon nanotubes (CNTs, Shenzhen Nanotech Port Co., Ltd.) was ultrasonically treated for 1 h in 50-mL nitric acid (HNO_3_, 68 wt%, Aladdin). The resulting suspension was heated at 120 °C for 12 h in a Teflon-lined autoclave. After thoroughly washed with deionized water, the HNO_3_-treated CNTs (0.25 g) were dispersed in 20 mL of aqueous solution of Mn(CH_3_COO)_2_·4H_2_O (1.69 g) with a 0.5-h ultrasonic treatment. Subsequently, the obtained solution was mixed with 80 mL of KMnO_4_ (0.727 g) aqueous solution and stirred for 0.5 h at room temperature. The resulting solution was then heated at 80 °C for 6 h under stirring. Finally, the obtained dark brown precipitate (denoted as CNT@MnO_2_) was washed several times by deionized water and dried at 80 °C in air for 12 h.

### Characterization Methods

#### Materials Characterizations

The X-ray diffraction (XRD) analysis was conducted by a Rigaku Mini Flex 600 diffractometer using Cu Kα radiation (*λ* = 1.5418). The scanning electron microscopy (SEM) images with corresponding energy-dispersive X-ray spectrometer (EDS) mappings were collected on a FESEM (FEI Nova NanoSEM 230, 10 kV). The crystallographic structures of the samples were identified using high-resolution transmission electron microscopy (HRTEM, Tecnai G2 F20). The XPS measurements were conducted by a ESCALAB 250 Xi X-ray photoelectron spectrometer. The content ratios of elements were investigated by inductively coupled plasma optical emission spectrometry (ICP-OES, Spectro Blue Sop).

#### Electrochemical Measurements

The potentiostatic charge–galvanostatic discharge performances were recorded using LAND battery cycler (CT2001A) at room temperature, in which the cells were charged and discharged at different current densities between 0.1 and 1 A g^−1^ the voltage region of 0.8 ~ 1.8 V. The cyclic voltammetry (CV) was tested on CHI660E at 0.1 mV s^−1^ from 0.8 ~ 1.8 V vs. Zn^2+^/Zn. Liner sweep voltammetry (LSV) was tested at 5 mV s^−1^.

#### Simulation of the Electric Field Contribution

A simplified 2D/3D electrodeposition model based on COMSOL Multiphysics software was established to compare the proportional schematics of electric field distribution and current density. The ionic conductivity of electrolyte was set as 5.0 S m^−1^.

### Animal Experiments

#### Animal Experiment

Animals were maintained in accordance with animal care guidelines established by the Laboratory Animal Ethics Committee of the Department of Laboratory Animals (CSU-2022-0122). Four months of age and weighing 2.5–3.0-kg male New Zealand white rabbits (*n* = 5, each group) were used in the current study. In brief, all operations were performed under general anesthesia with 30 mg kg^−1^ pentobarbital sodium. The batteries are employed in this work after punching with 1-mm diameter.

#### Assess Battery-Related Injuries

Rabbits were shaved under general anesthesia. We choose an abdominal “T-type” incision to exposure the entire stomach, a median abdominal incision to exposure the colon, and a hind thigh incision to exposure the skeletal muscle. The cathode side of the battery was placed on the mucosal surface or the muscle surface for 6 h. Lifting the batteries to expose the tissue and photographed every 2 h, then returning it to its original place. Tissues were collected after 6 h and then stored in 10% formalin. The hematoxylin and eosin (HE) staining was performed to assess the injury degree.

#### Battery Implantation into Rabbits

Rabbits were shaved under general anesthesia. The battery was implanted into the subcutaneous space through a 2-cm incision. After implantation, the incisions were closed using 4–0 silk suture. After 60 days of implantation, the implants and the surrounding implant capsules were extracted. The inflammatory response and compatibility of the implants were assessed by the HE staining and Masson’s trichrome (M and T) staining. The thickness of implant capsules and the collagen density was evaluated by ImageJ.

## Results and Discussion

**Fig. 2:**
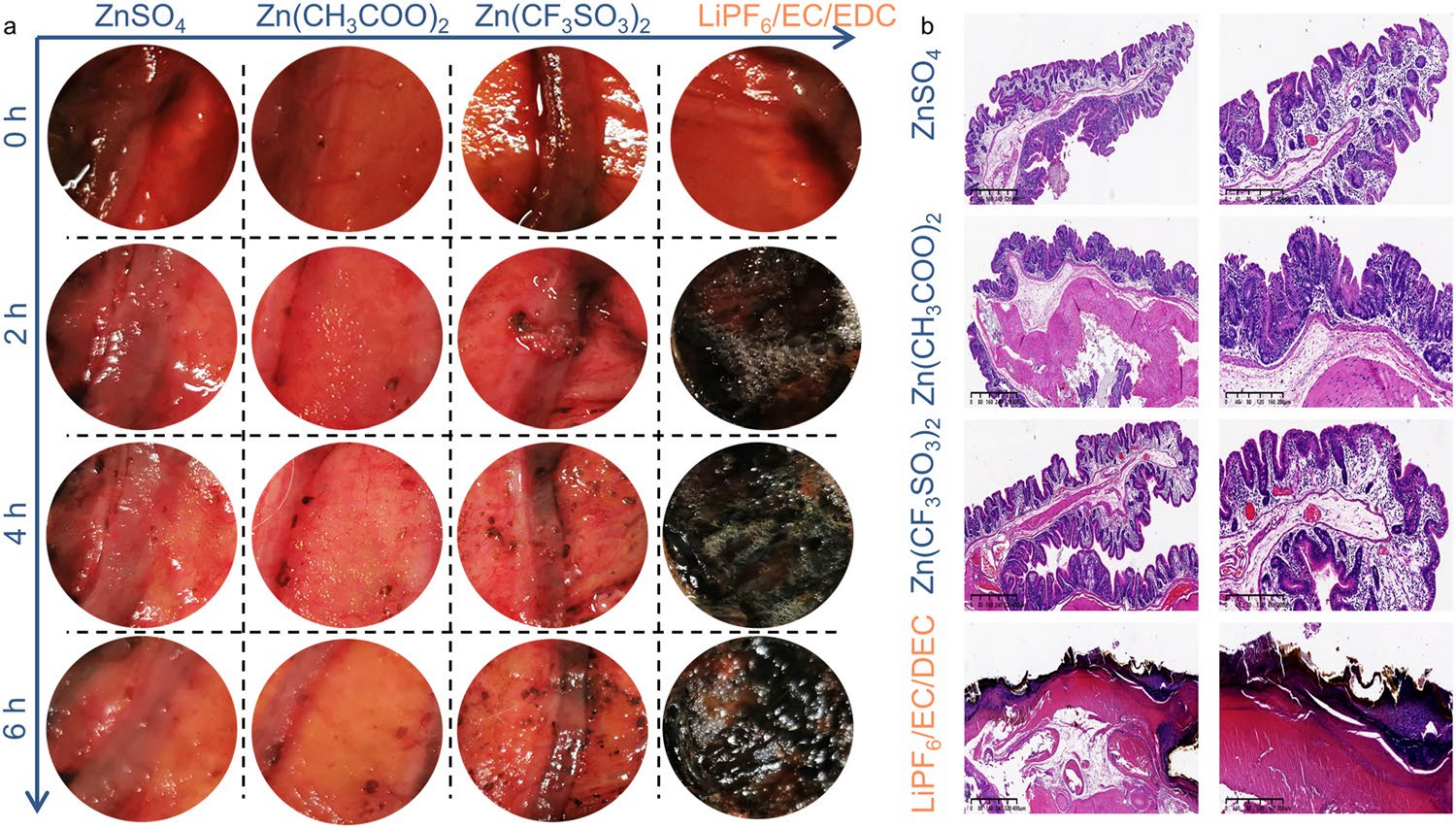
**a** Leakage scene simulation tests on the colon mucosa and **b** the corresponding HE staining results

Generally, battery biocompatibility research includes the investigations on the operating stability and biosecurity when considering the battery applications in wearable and implantable devices [37]. Biocompatible ZIBs are known to apply flexible configurations to keep operating stability in some cases while their biosecurity is promised with high-safety components, including cathode, anode, electrolyte, and separator materials [32]. For ZIBs, most of the components are of great stability and safety [38]; thus, the battery biosecurity is mainly determined by the aqueous electrolytes, especially the zinc salts therein. Aqueous ZIBs based on different electrolytes are tested on the living rabbit models to determine the histocompatibility, while the Li-ion batteries are used as controls. The initial visible changes start to occur on the sample with Li-ion battery in 2 h. Gas bubbles with circular and brown discoloration around the contact surfaces of the Li-ion battery are observed in colon wall (Fig. 2a), gastric wall (Fig. S1), and skeletal muscle of hind limb (Fig. S2). After 6 h, there is significant blackening and burning in the gastric mucosa, colonic mucosa, and muscle surface around the Li-ion battery. There is little progression in the visible changes observed for the Zn(CF_3_SO_3_)_2_ and Zn(CH_3_COO)_2_ batteries and less so for the ZnSO_4_ batteries. The hematoxylin and eosin (HE) staining technique is extensively used to display the general morphological and structural characteristics of various tissues or cell components and lesions. Here, HE-stained sections present significantly greater depths of necrotic tissue in the control than in these three ZIBs groups (Fig. 2b), among which ZnSO_4_ exhibits the minimal destruction.

**Fig. 3:**
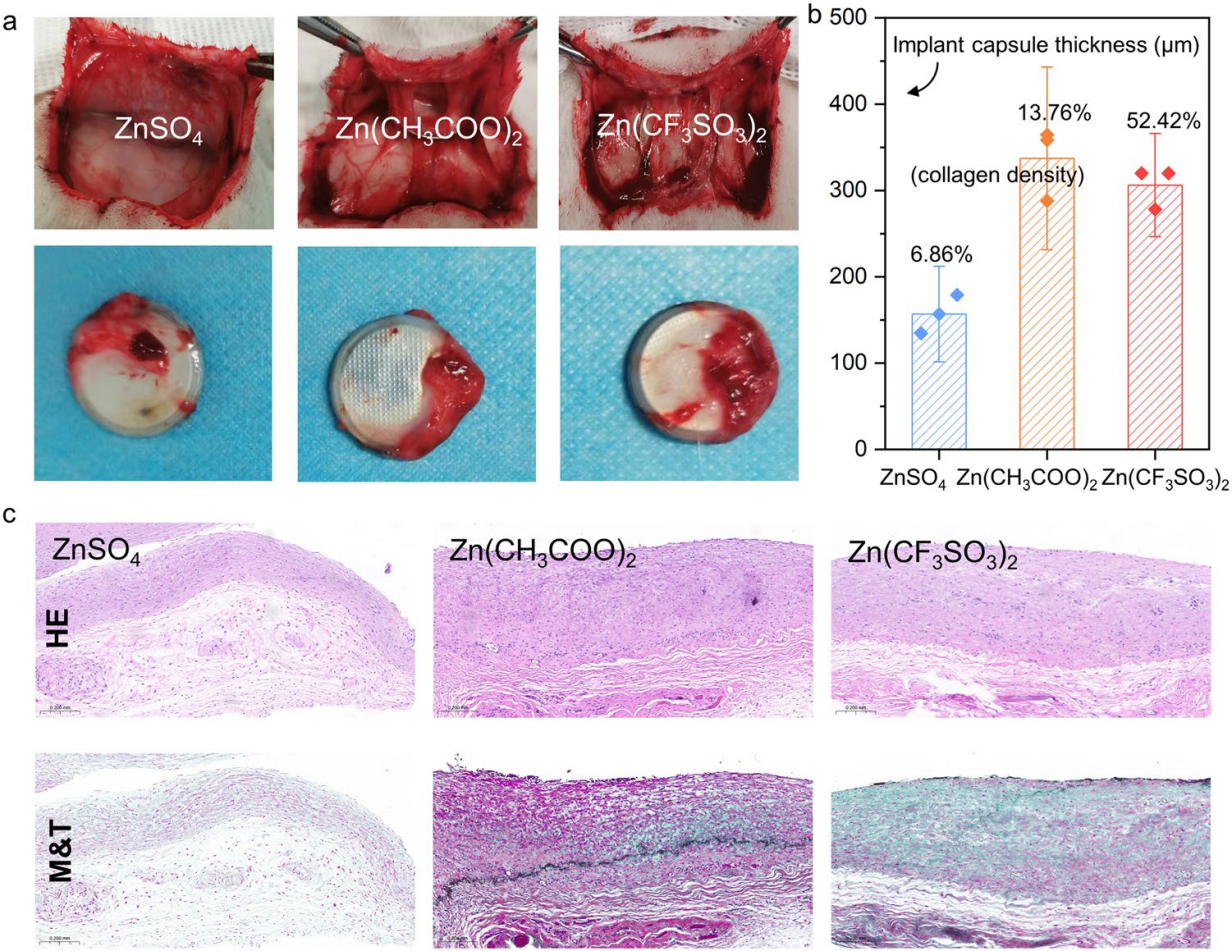
**a** Optical images of the implant capsules of three ZIBs. **b** Thickness of the implant capsules and the corresponding quantified data of collagen density of the batteries–tissue interface. **c** HE staining and M and T staining results of the collagen encapsulation 2 months after the Zn-based battery implantations. Muscle fibers (red), collagen fibers (green–blue), and nuclei (dark purple). (Color figure online)

Additionally, coin cells based on different electrolytes are implanted in the dorsal subcutaneous region of rabbits for 60 days (Fig. S3a). After 2 weeks, the region with Li-ion battery implanted develops a sore that hardly heals, and the purulent drainage draining from the wound is observed (Fig. S3b). We evaluate the tissues surrounding three ZIBs after the implantation and find that the tissues surrounding implanted region are mild swelling after 1 week, and full recovery covering with fur from implanting operation is achieved within 30 days (Fig. S4). After 60 days of implantation, the HE-stained sections show that the structure of the implantation regional skin turns to be normal without inflammatory cell infiltration among Zn(CF_3_SO_3_)_2_, Zn(CH_3_COO)_2_, and ZnSO_4_ batteries (Fig. S5). These three ZIBs are surrounded by the thin implant capsules of few inflammatory cells and collagen (Fig. 3a), exhibiting the thickness of 336 μm for Zn(CH_3_COO)_2,_ 306 μm for Zn(CF_3_SO_3_)_2_, and 157 μm for ZnSO_4_ (Fig. 3b). The collagen encapsulation of ZnSO_4_ batteries is significantly thinner than that of Zn(CF_3_SO_3_)_2_ and Zn(CH_3_COO)_2_ batteries, which implies that ZnSO_4_ exhibits superior biosecurity than Zn(CF_3_SO_3_)_2_ and Zn(CH_3_COO)_2_, regarding compatibility to the host. One of the staining methods used to display fibers and inflammatory factors in tissues. Masson’s trichrome (M and T) is one of the staining methods used to display fibers and inflammatory factors in tissues. Figure 3c presents the HE staining and M and T staining images of the collagen encapsulation of batteries based on ZnSO_4_, Zn(CH_3_COO)_2_, and Zn(CF_3_SO_3_)_2_, of which the collagen density at the interface is 6.86%, 13.76%, and 52.42%, respectively. These findings imply that ZnSO_4_ is superior to Zn(CF_3_SO_3_)_2_ and Zn(CH_3_COO)_2_ batteries when regarding the electrolyte biocompatibility for ZIBs.

**Fig. 4:**
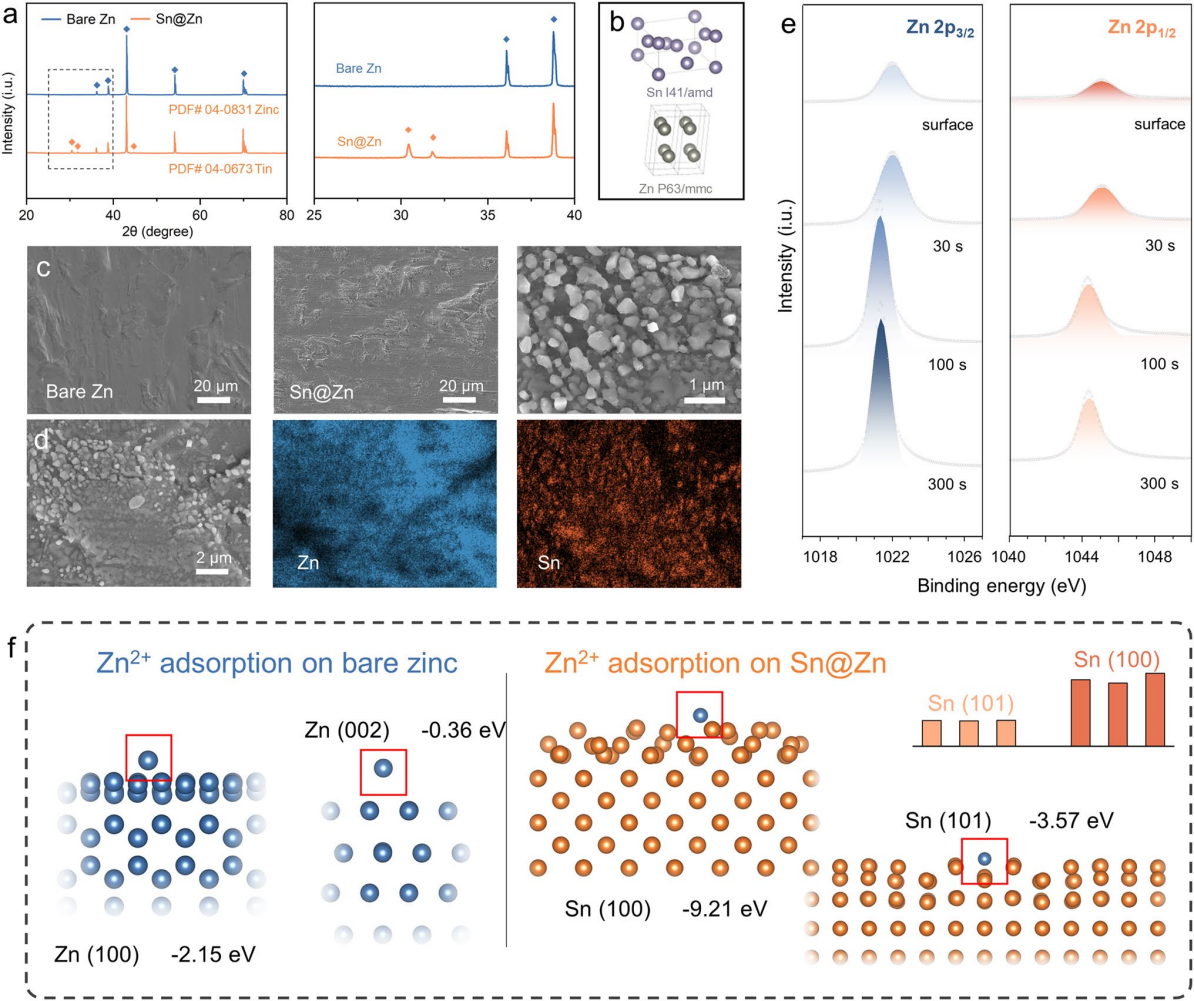
Morphological and structural investigations on the heterogenous layer. **a** XRD spectra of bare zinc and Sn@Zn foil. **b** Schematics of the crystal structures of Sn, Zn, and Sn@Zn. **c** SEM images of bare zinc and Sn@Zn foil. **d** Elemental mapping results of Sn@Zn foil. **e** Depth-dependent Zn 2*p* XPS spectra of Sn@Zn foil. **f** Calculated models of Zn^2+^ adsorbed on Zn (100), Zn (100), Sn (100), and Sn (101)

Although ZnSO_4_ is proved to be of high biosecurity, the safety issues during battery operating should also be considered. Battery inflation and short circuit induced by notorious dendrite growth and hydrogen evolution in mildly acidic ZnSO_4_ electrolyte are threatening the development of biocompatible ZIBs [39]. To mitigate these anode issues, we introduce the Sn hetero nucleus on the anode zinc foil, which is obtained by simply immersing a zinc foil in the solution of 0.01 M SnSO_4_ for 1 min. As shown in the XRD spectra (Fig. 4a), relatively weak signals of Sn are detected on the Sn@Zn foil, indicating the existence of Sn element in minute amounts (Fig. 4b). Furthermore, the morphology of Sn@Zn foil is presented in the SEM images in Fig. 4c, which proves that constructing the heterogenous layer causes neglectable changes to the zinc foil. It is worth noting that the heterogenous layer is formed as the nanoscale Sn particles generated on the zinc foil (Fig. 4d), which allows the efficient Zn plating/stripping through the interspace. Similar results have been obtained by conducting the depth-dependent XPS spectra of Sn@Zn foil with the etching rate of 6 nm s^−1^. As shown in Fig. 4e, the Zn 2*p* signals become stronger in deeper positions, while the opposite situation is observed in Sn 3*p* signals (Fig. S6). The structure models explaining Zn^2+^ adsorption on different Zn planes and Sn planes are shown in Fig. 4f. The calculated adsorption energy of Zn^2+^ on Zn (100) and Zn (002) is − 2.15 and − 0.36 eV, which are much higher than − 9.21 and − 3.57 eV on Sn (100) and Sn (101), indicating that Zn^2+^ is preferentially adsorbed on Sn surface.

**Fig. 5:**
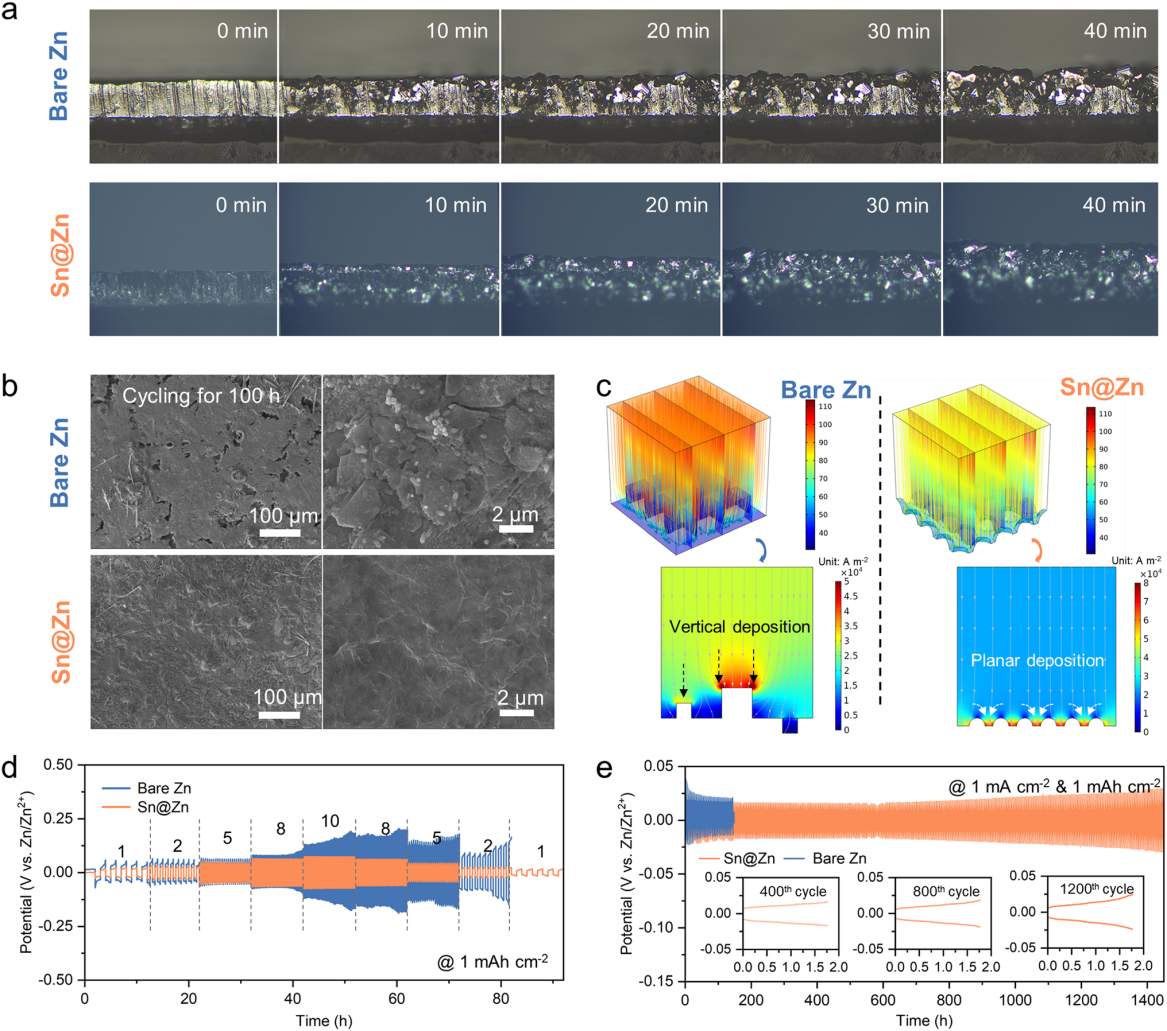
Zn plating/stripping process on the Sn@Zn foil. **a** In situ optical microscopy observation of the zinc deposition process on bare Zn and Sn@Zn foil. **b** SEM images of bare Zn and Sn@Zn foil after cycling for 100 h. **c** Finite element simulation results of zinc deposition process on bare Zn and Sn@Zn foil. Herein, the blocks are representing the disordered pits and piles for bare Zn foil, and the semicircles indicate the hetero Sn nucleus. **d** Rate capability tests of the symmetrical cells based on bare Zn and Sn@Zn foil. **e** Long cycling tests of the symmetrical cells based on bare Zn and Sn@Zn foil

To investigate the Zn electrochemical behaviors on the Sn@Zn foil, the Zn deposition process is firstly visualized under the optical microscopy observation (Fig. 5a). In the electrolyte of 2 M ZnSO_4_, obvious Zn deposition occurs on both the Zn foil and Sn@Zn foil in 40 min, of which the former turns to be uneven while Zn is uniformly deposited on the Sn@Zn foil. To further study the Zn plating/stripping process, SEM images of the foils after cycling for 100 h have been captured (Fig. 5b). The morphology of Zn foil presents to be disordered with pits and piles, indicating the uneven deposition process and the risk of dendrite growth. The morphology of Sn@Zn foil after cycling is much smoother without obvious deficiency, which should be attributed to the facilitated planar Zn deposition by the hetero nucleus growth [40], as supported by the EIS spectra of symmetrical cells (Fig. S7). The finite element simulation results presented in Fig. 5c indicate that the uneven morphology of Zn foil will lead to uneven electrical field distribution and exaggerated dendrite growth, while the Sn nucleus contributes to the smooth deposition by increasing the local current density in the interspace. Except for regulating the Zn deposition process, this heterogenous layer also contributes to anode protection with the higher hydrogen evolution overpotential of Sn particles. As shown in Fig. S8a, the LSV curves with the scanning rate of 5 mV s^−1^ indicate severer hydrogen evolution on bare Zn foil (Fig. S8b), while the larger corrosion current density (4.6 mA cm^−2^ compared with 1.284 mA cm^−2^ for Sn@Zn foil) delivers the same results. Based on the above merits of introducing Sn nucleus, the symmetrical cell based on Sn@Zn foil exhibits much lower polarization voltage gap (Fig. S9) under different current densities (from 1 to 10 mA cm^−2^) and better stability during the rate capability tests (Fig. 5d). Thanks to the regulated Zn deposition (Fig. S10), the symmetrical cell based on Sn@Zn foil achieves the long lifetime of 1500 h under the cycling protocols of 1 mA cm^−2^ and 1 mAh cm^−2^ (Fig. 5e), which indicates much better stability than bare Zn foil (Fig. S11).

**Fig. 6:**
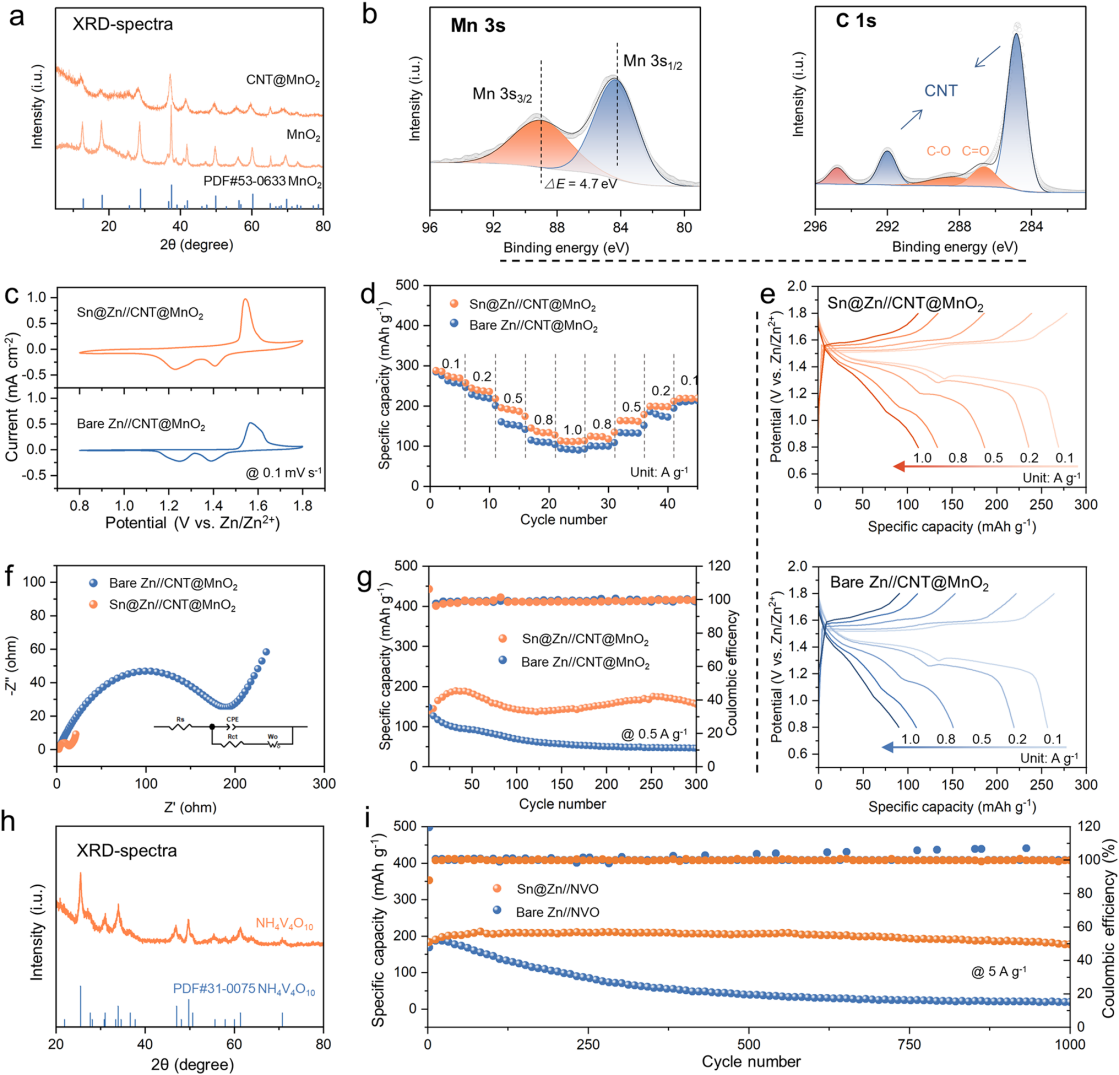
Electrochemical performance of Zn–MnO_2_ batteries based on the Sn@Zn foil. **a** XRD spectra of the synthesized MnO_2_ and CNT@MnO_2_. **b** Mn 3*s* and C 1*s* XPS spectra of the synthesized CNT@MnO_2_. **c** CV curves of the Zn–MnO_2_ batteries based on bare Zn and Sn@Zn foil. **d** Rate capability tests of the Zn–MnO_2_ batteries based on bare Zn and Sn@Zn foil and **e** the corresponding charge–discharge profiles. **f** Fitted EIS spectra and **g** long cycling performance of the Zn–MnO_2_ batteries based on bare Zn and Sn@Zn foil. **h** XRD spectra of the synthesized NVO. **i** The corresponding cycling performance of Zn-NVO batteries based on bare Zn and Sn@Zn foil

To explore the electrochemical capability of full ZIBs, CNT@MnO_2_ is coupled with Sn@Zn and bare Zn foil, respectively. As shown in the XRD spectra (Fig. 6a), diffractions peaks of the synthesized MnO_2_ are indexed to PDF#53-0633 [41], of which the crystal structure remains unchanged after compositing with the highly conductive CNT powders. Meanwhile, Mn 3*s* and C 1*s* XPS spectra present supportive results. As shown in Fig. 6b, the separation energy of 4.7 eV in Mn 3*s* spectra proves the + 4 valence in synthesized MnO_2_, while the signals of C–O and C=O in C 1*s* should be attributed to the hydrophilic treatment to CNT [42]. Based on this CNT@MnO_2_ cathode, CV curves are collected as presented in Fig. 6c, which indicate that introducing Sn nucleus causes no side reactions to the Zn–MnO_2_ battery system. Subsequently, the rate capability of Zn–MnO_2_ batteries based on different anodes has been tested under varying current densities (from 0.1 to 1 A g^−1^), of which the corresponding charge–discharge profiles are collected. As shown in Fig. 6d, the battery based on Sn@Zn anode exhibits higher specific capacity (280 mAh g^−1^ under 0.1 A g^−1^) especially under large current density, indicating better rate capability (Fig. 6e). These results should be attributed to the facilitated Zn deposition on Sn@Zn foil, as supported by the EIS spectra of Zn–MnO_2_ batteries based on different anodes (Fig. 6f). Ultimately, owing to the superiority of Sn@Zn anode, the Sn@Zn-CNT@MnO_2_ battery achieves the prolonged lifetime for 300 cycles under 0.5 A g^−1^ (Fig. 6g), presenting higher capacity retention and better cycling stability than the battery based on bare Zn anode. NH_4_V_4_O_10_ (NVO) is also applied as the cathode materials, of which the XRD spectra and SEM images are presented in Figs. 6h and S12. As shown in Fig. 6i, the Zn-NVO batteries based on Sn@Zn anode present higher specific capacity and capacity retention after 500 cycles. Therefore, a stabler lifetime of 1000 cycles (212 mAh g^−1^) under 5 A g^−1^ is obtained with neglectable capacity degradation.

## Conclusions

In this paper, we devolep three ZIBs that have the excellent stability with high biosecurity, especially ZnSO_4_. No obvious tissue damage occurs within 6 h after exposure to these ZIBs; however, significant damage occurs in as little as 2 h after exposure to Li-ion batteries. The in vivo implantation experiments demonstrated that these three ZIBs are non-toxic, non-allergenic, and not elicit excessive tissue and immune responses. The ZIBs implants are resistant to corrosion when exposed to bodily fluids. The above-mentioned characteristics are necessary for chronic implantation. Furthermore, Sn hetero nucleus has been introduced on the zinc foil surface, facilitating zinc deposition and mitigating hydrogen evolution, thus stabilizing Zn anode during cycling. As a result, the Sn@Zn symmetrical cells achieve a long lifetime of 1500 h. When coupled with the CNT@MnO_2_ cathode, the battery exhibits the specific capacities of 280 mAh g^−1^ under 0.1 A g^−1^. The Sn@Zn-NVO batteries exhibit a long lifetime of 1000 cycles (212 mAh g^−1^) under 5 A g^−1^. This work provides a novel view from evaluating the biosecurity of electrolyte toward biocompatible ZIBs. Except for the above results, relevant battery components may also be considered for their biosecurity and operating stability in the future, including exploiting high-safety package materials, stable Zn anode, and rational design of hydrogel electrolyte matrix, etc.

### Supplementary Information

Below is the link to the electronic supplementary material.

Supplementary file1 (PDF 1103 KB)
