# Supplementary material for: Hetero Nucleus Growth Stabilizing Zinc Anode for High-Biosecurity Zinc-Ion Batteries
Source: Nanomicro Lett. 2023 Oct 26;15:237. doi: 10.1007/s40820-023-01206-2 (PMC10603014; doi:10.1007/s40820-023-01206-2)
Supplement: Supplementary file 1 — Supplementary file1 (PDF 1103 KB) [file 40820_2023_1206_MOESM1_ESM.pdf]

Supporting Information for

Hetero Nucleus Growth Stabilizing Zinc Anode for High-Biosecurity Zinc-Ion Batteries

Jingjing Li[1], Zhexuan Liu[2], Shaohua Han[2], Peng Zhou[3], Bingan Lu[4], Jianda Zhou[5], Zhiyuan Zeng[6], Zhizhao Chen[1, 5, *], and Jiang Zhou[2, *]

[1] Department of Plastic Surgery and National Clinical Research Center for Geriatric Disorders, Xiangya Hospital, Central South University, Changsha 410008, P. R. China

[2] School of Materials Science and Engineering, Hunan Provincial Key Laboratory of Electronic Packaging and Advanced Functional Materials, Central South University, Changsha 410083, P. R. China

[3] Hunan Provincial Key Defense Laboratory of High Temperature Wear-Resisting Materials and Preparation Technology, Hunan University of Science and Technology, Xiangtan, 411201, P. R. China

[4] School of Physics and Electronics, Hunan University, Changsha, 410082, P. R. China

[5] Department of Plastic Surgery, The Third Xiangya Hospital, Central South University, Changsha, 410013, P. R. China

[6] Department of Materials Science and Engineering, City University of Hong Kong 83 Tat Chee Avenue, Kowloon, Hong Kong, 999077, P. R. China

*Corresponding authors. E-mail: czz657033698@126.com (Zhizhao Chen); zhou_jiang@csu.edu.cn (Jiang Zhou)

S1 Experimental Section

Materials: All the reagents are of analytical purity and used as received without further purification. Zinc sulfate heptahydrate ($ZnSO_4 \cdot 7H_2O$, ≧ 99%), stannous sulfate ($SnSO_4$, ≧ 98%), manganese sulfate ($MnSO_4$, ≧ 99%), zinc acetate tetrahydrate ($Zn(CH_3COO)_2 \cdot 4H_2O$, ≧ 99%), zinc trifluoromethanesulfonate ($Zn(CF_3SO_3)_2$, ≧ 99%), lithium hexafluorophosphate ($LiPF_6$/EC, 5%).

Construction of Sn@Zn foil: 0.01M $SnSO_4$ solution is obtained by dissolving $SnSO_4$ into deionized water and string for 30 min. Bare Zn foil is cut into needed circular sheets (15 mm diameter) and immersed in 0.01M $SnSO_4$ solution for 1 min. The obtained Sn@Zn foil is washed with deionized water for several times and dried at 80 °C in air for 12 h.

Synthesis of CNT@MnO$_2$: 1.5 g multiwalled carbon nanotubes (CNTs, Shenzhen Nanotech Port Co., Ltd.) was ultrasonically treated for 1 h in 50 mL nitric acid (HNO_3, 68 wt %, Aladdin). The resulting suspension was heated at 120 °C for 12 h in a Teflon-lined autoclave. After thoroughly washed with deionized water, the HNO_3-treated CNTs (0.25 g) were dispersed in 20 mL of aqueous solution of $Mn(CH_3COO)_2 \cdot 4H_2O$ (1.69 g) with a 0.5 h ultrasonic treatment. Subsequently, the obtained solution was mixed with 80 mL of $KMnO_4$ (0.727 g) aqueous solution and stirred for 0.5 h at room temperature. The resulting solution was then heated at 80 °C for 6 h under stirring. Finally, the obtained dark brown precipitate (denoted as CNT@MnO_2) was washed several times by deionized water and dried at 80 °C in air for 12 h.

Materials Characterizations: The X-ray diffraction (XRD) analysis were conducted by a Rigaku Mini Flex 600 diffractometer using Cu Kα-radiation (λ= 1.5418). The scanning electron microscopy (SEM) images with corresponding energy dispersive X-ray spectrometer (EDS) mappings were collected on a FESEM (FEI Nova NanoSEM 230, 10 kV). The crystallographic structures of the samples were identified using high-resolution transmission electron microscopy (HRTEM, Tecnai G2 F20). The XPS measurements were conducted by a ESCALAB 250 Xi X-ray photoelectron spectrometer. The content ratios of elements were investigated by inductively coupled plasma optical emission spectrometry (ICP-OES, Spectro Blue Sop).

Electrochemical measurements: The potentiostatic charge-galvanostatic discharge performances were recorded using LAND battery cycler (CT2001A) at room temperature, in which the cells were charged and discharged at different current densities between 0.1 to 1 A g^{-1} the voltage region of 0.8 ~ 1.8 V. The Cyclic Voltammetry (CV) was tested on CHI660E at 0.1 mV s^{-1} from 0.8 ~ 1.8 V *vs.* Zn^{2+}/Zn. Liner sweep voltammetry (LSV) was tested at 5 mV s^{-1}.

Simulation of the electric field contribution: A simplified 2D/3D electrodeposition model based on COMSOL Multiphysics software was established to compare the proportional schematics of electric field distribution and current density. The ionic conductivity of electrolyte was set as 5.0 S m^{-1}.

Animal Experiment: Animals were maintained in accordance with animal care guidelines established by the Laboratory Animal Ethics Committee of Department of laboratory animals (CSU-2022-0122). Four months of age and weighing 2.5-3.0 kg male New Zealand white rabbits (n = 5, each group) were used in the current study. In brief, all operations were performed under general anesthesia with 30 mg/kg pentobarbital sodium. The batteries are employed in this work after punching with 1 mm diameter.

Assess battery-related injuries: Rabbits were shaved under general anesthesia. We choose an abdominal "T type" incision to exposure the entire stomach, a median abdominal incision to exposure the colon, and a hind thigh incision to exposure the skeletal muscle. The cathode side of the battery was placed on the mucosal surface or the muscle surface for 6 hours. Lifting the batteries to expose the tissue and photographed every two hours, then returning it to its original place. Tissues were collected after 6 hours and then stored in 10% formalin. The hematoxylin & eosin (HE) staining was performed to assess the injury degree.

Battery implantation into rabbits: Rabbits were shaved under general anesthesia. The battery was implanted into the subcutaneous space through a 2-cm incision. After implantation, the incisions were closed using 4-0 silk suture. After 60 days of implantation, the implants and the surrounding implant capsules were extracted. The inflammatory response and compatibility of the implants were assessed by the HE staining and Masson's trichrome (M&T) staining. The thickness of implant capsules and the collagen density was evaluated by Image J.

Adsorption energy calculation: Vienna ab initio simulation package (VASP) was employed to perform all density functional theory (DFT) calculations within the generalized gradient approximation (GGA), which uses the Perdew-Burke-Ernzerhof (PBE) formulation. We have selected the projected augmented wave (PAW) potentials to describe the ionic cores. Additionally, we take valence electrons into account using a plane wave basis set with a kinetic energy cutoff of 500 eV. Partial occupancies of the Kohn−Sham orbitals were allowed under the occasion which uses the Gaussian smearing method and a width of 0.03 eV. The electronic energy can be considered self-consistent if the energy change was smaller than 10^{-8} eV. When the force change is less than 0.02 eV/Å, the geometric optimization is considered to converge. The dispersion interactions were described by Grimme's DFT-D4 methodology. Uniform G-centered k-points meshes with a resolution of $2\pi*0.04$ Å^{-1}. The adsorption energy (E_{ads}) of adsorbate A was defined as

$$E_{ads} = E_{A/surf} – E_{surf} - E_A(g)$$

where $E_{A/surf}$, E_{surf} and $E_A(g)$ are corresponding to the energy of adsorbate A adsorbed on the surface, the energy of clean surface, and the energy of isolated A molecule in a cubic periodic box, respectively.

S2 Supplementary Figures and Tables

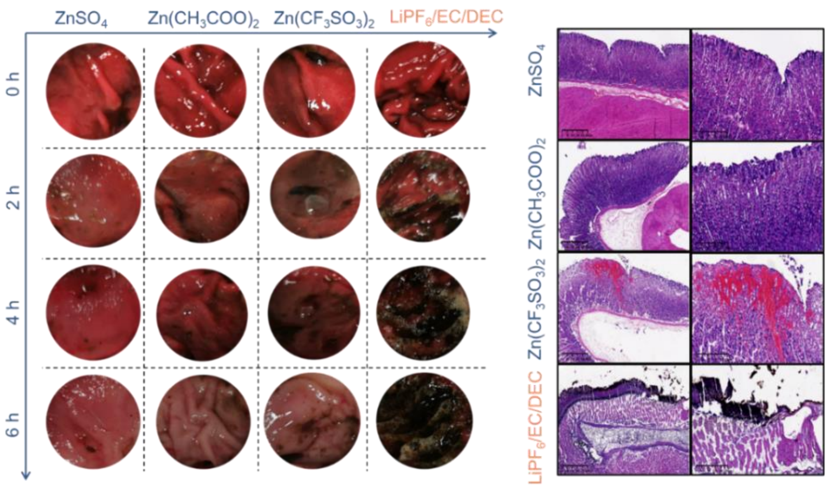

Fig. S1 *In-situ* optical images and HE staining results of the gastric mucosa after battery leakage scene simulations

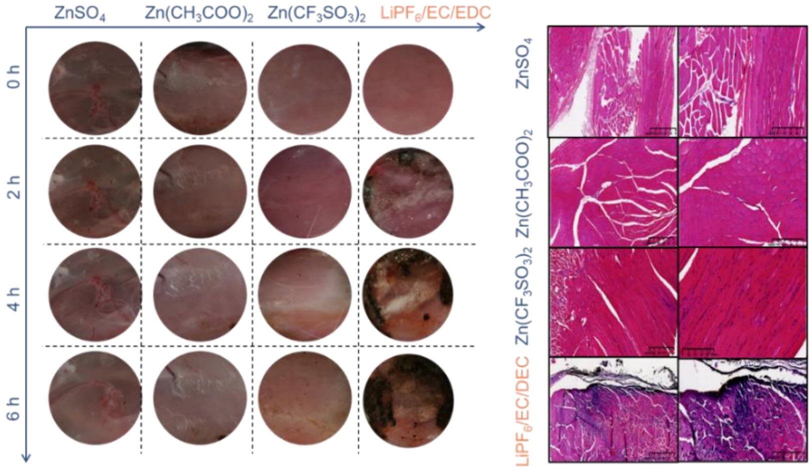

Fig. S2 *In-situ* optical images and HE staining results of the thigh muscle after battery leakage scene simulations

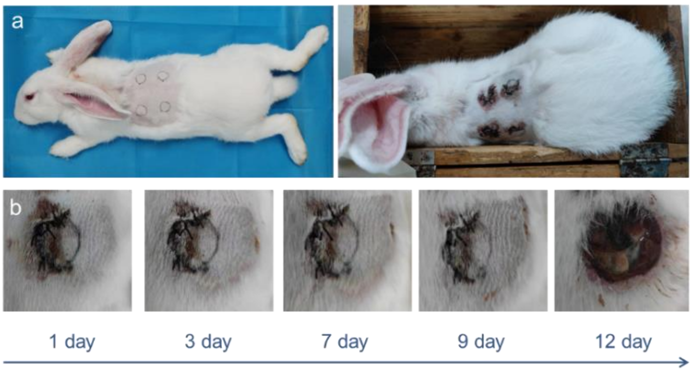

Fig. S3 (**a**) Photographs of the experimental rabbit for battery implantation. (**b**) *In-situ* optical images of the subcutaneous area after the Li-based battery implantation

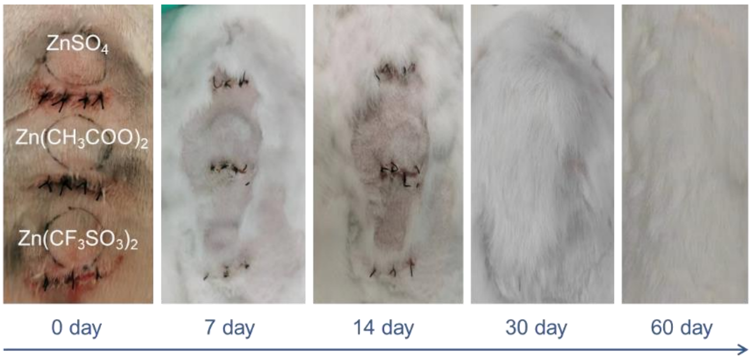

Fig. S4 *In-situ* optical images of the subcutaneous area after the Zn-based battery implantations

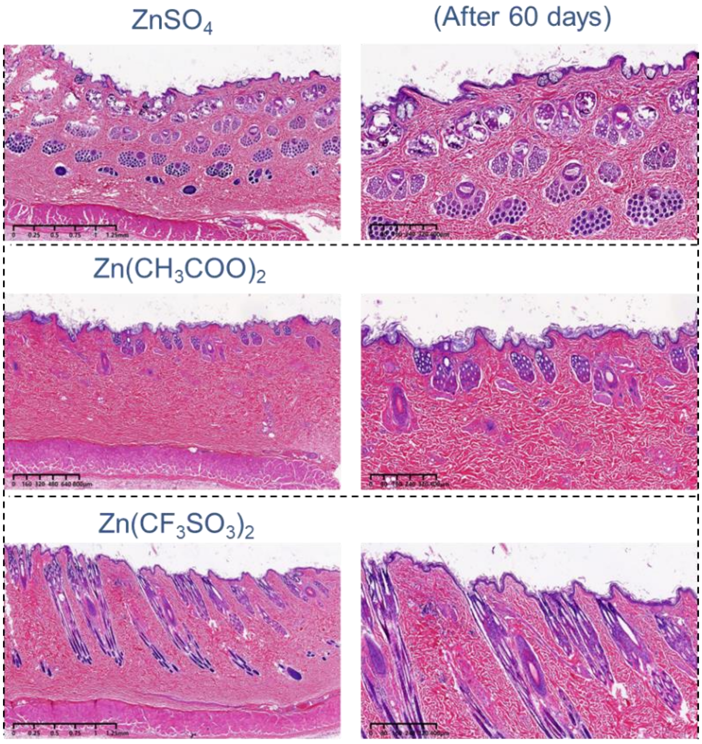

Fig. S5 HE staining results of the subcutaneous area after battery implantations for 60 days

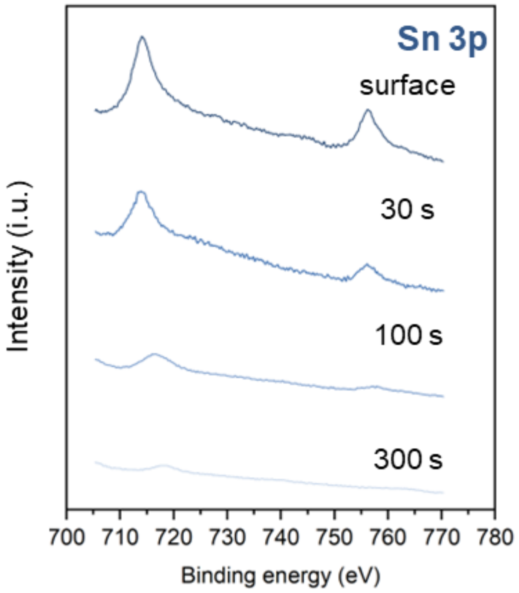

Fig. S6 Depth-dependent Sn 3p XPS spectra of Sn@Zn foil

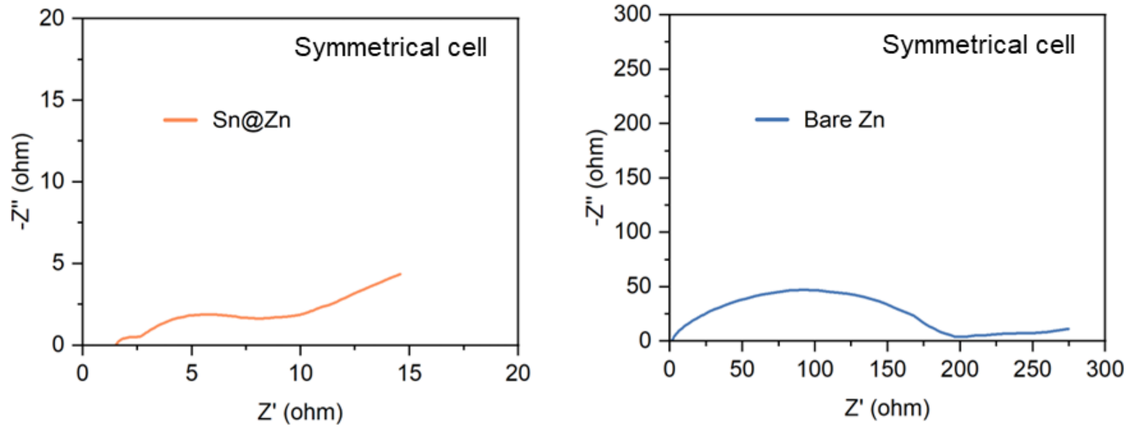

Fig. S7 EIS spectra of the symmetrical cells based on different anodes

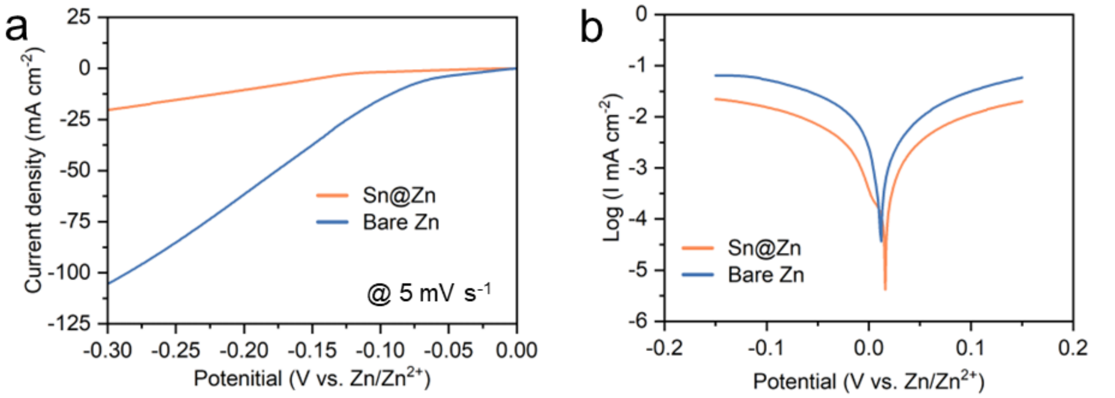

Fig. S8 (**a**) LSV analysis of hydrogen evolution on Sn@Zn foil and bare Zn foil. (**b**) Tafel spectra of Sn@Zn foil and bare Zn foil

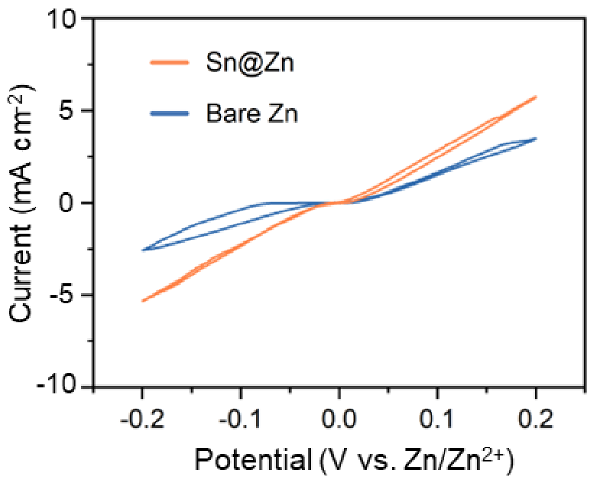

Fig. S9 CV curves of the symmetrical cells based on different anode foils

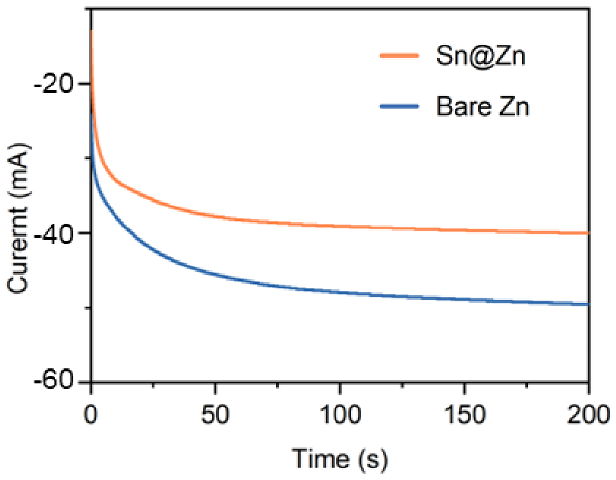

Fig. S10 Chronoamperometry (CA) curves of different anode foils

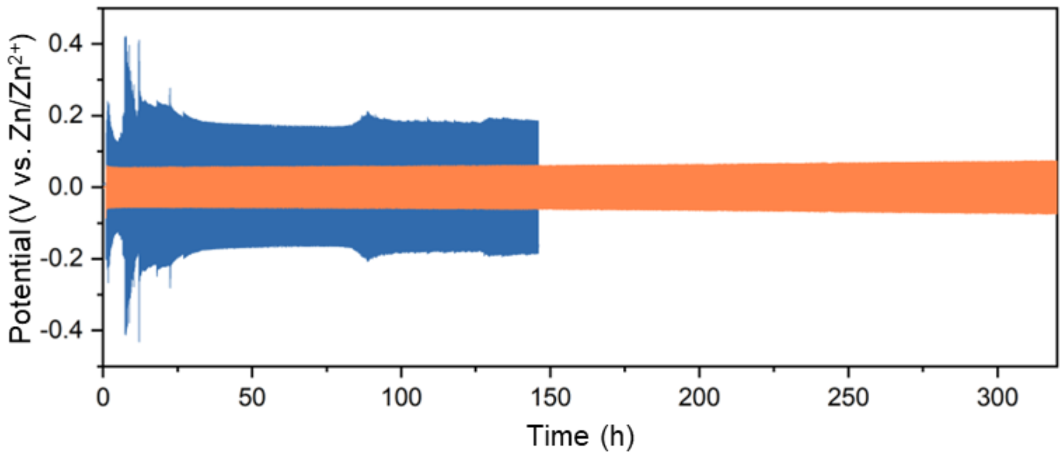

Fig. S11 Long cycling tests of the symmetrical cells based on bare Zn and Sn@Zn foil under 10 mA cm^{-2}

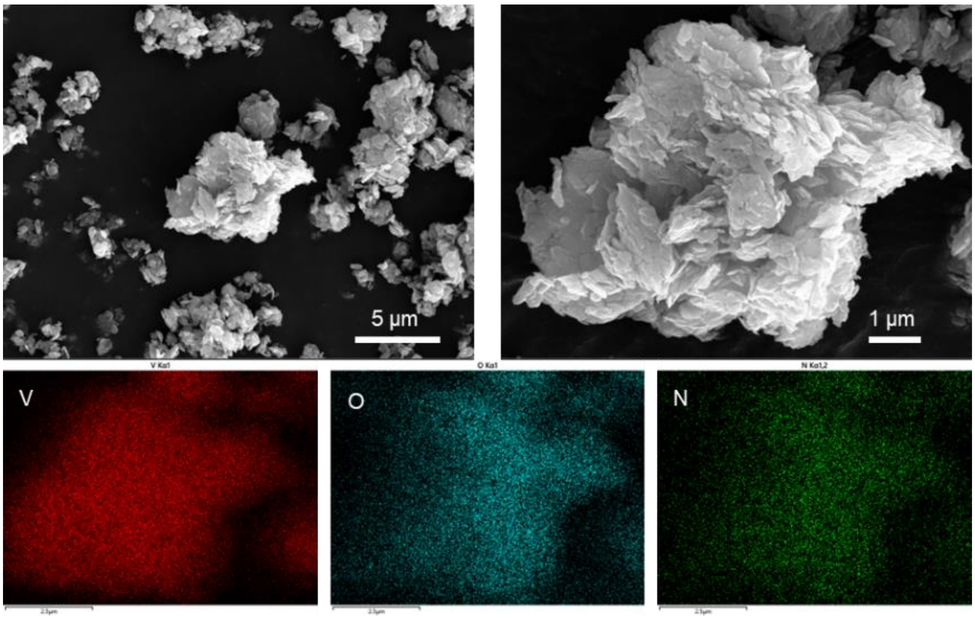

Fig. S12 SEM images and the corresponding elemental mapping images of the synthesized NVO
